# Relationship Between Social Risk Factors and Emergency Department Use: National Health Interview Survey 2016–2018

**DOI:** 10.5811/westjem.18616

**Published:** 2024-12-31

**Authors:** Iraa Guleria, Jennifer A. Campbell, Abigail Thorgerson, Sanjay Bhandari, Leonard E. Egede

**Affiliations:** * Medical College of Wisconsin, Center for Advancing Population Science, Milwaukee, Wisconsin; † University at Buffalo, State University of New York, Jacobs School of Medicine and Biomedical Sciences, Department of Medicine, Division of Population Health, Buffalo, New York; ‡ Medical College of Wisconsin, Department of Medicine, Division of General Internal Medicine, Milwaukee, Wisconsin; § University at Buffalo, State University of New York, Jacobs School of Medicine and Biomedical Sciences, UBMD Internal Medicine, Buffalo General Medical Center, Buffalo, New York

## Abstract

**Background:**

Evidence shows that social risks are highly prevalent in the patient population that presents to the emergency department (ED) for care; however, understanding the relationship between social risk factors and ED utilization at the population level remains unknown.

**Methods:**

We used the National Health Interview Survey from the 2016–2018 sample adult files. The sample included 82,364 individuals, representing a population size of 238,888,238. The primary independent variables included six social risk factors: economic instability; lack of community; educational deficit; food insecurity; social isolation; and inadequate access to care. The outcome included ED use in the prior year. Covariates included age, race/ethnicity, insurance status, obesity, mental health (depression/anxiety), and comorbidities. We ran logistic regression models to test the relationship between the independent and dependent variables adjusting for covariates.

**Results:**

In the study sample, 20% had at least one ED visit in the prior year. In the fully adjusted model, individuals reporting economic instability (odds ratio [OR] 1.33, 95% confidence interval [CI] 1.25-1.42), lack of community (OR 1.10, 95% CI 1.05-1.15), educational deficit (OR 1.12, 95% CI 1.06-1.18), food insecurity (OR 1.77, 95% CI 1.66-1.89), and social isolation (OR 1.32, 95% CI 1.26-1.39) had significantly higher odds of ED use. Inadequate access to care was significantly related to lower odds of ED use (OR 0.75, 95% CI 0.69-0.81).

**Conclusions:**

Social risk factors are significantly associated with higher odds of ED use in the United States adult population. Interventions that integrate social and medical needs are greatly needed, as is understanding the role that preventive medicine may play in reducing avoidable ED visits.

Population Health Research Capsule What do we already know about this issue?

*Social risk factors play a role in access to healthcare. Prevalence of multiple social risk factors and their association with ED use at a national level remains unclear.*

What was the research question?

*What is the relationship between multiple social risk factors and ED use in a nationally representative sample of US adults?*

What was the major finding of the study?

*Economic instability, lack of community, educational deficit, food insecurity, and isolation increased (*P* < 0.001) ED use.*

How does this improve population health?

*These results provide targets for intervention development and clinical screening programs to reduce unnecessary ED use and healthcare costs.*

## INTRODUCTION

Emergency department (ED) use in the United States remains high, with the most recent national estimates showing 18% of US adults had at least one visit in the prior 12 months.^1^ ^–^ ^3^ Cost of ED utilization has remained over $76 billion per year,^4^ with an estimated $30 billion spent on preventable hospitalizations.^4^ Although the primary role of an ED is providing medical services to high-acuity or life-threatening conditions, overuse and misuse of the ED in the US remains a major concern for population health, with increased attention being given to the role of unmet social needs underlying ED utilization.^5^ ^,^ ^6^

It has been well-established that an individual’s social and physical environment plays a role in health outcomes and is becoming increasingly important for understanding access to health services.^7^ ^–^ ^11^ Evidence shows that social risk factors, the adverse social conditions such as economic instability, food and housing instability, and limited access to transportation, are highly prevalent among patients who present to the ED.^6^ ^,^ ^12^ ^–^ ^19^ For example, lower socioeconomic status and poverty are associated with increased ED and emergency service utilization rates,^16^ ^–^ ^18^ with evidence showing that those presenting to the ED are more likely to be low income^19^ and insured through Medicaid.^20^ At the national level, food insecurity is independently associated with a 47% higher ED utilization rate.^14^ Similarly, individuals with housing insecurity have a two-fold increased risk of ED use,^15^ with a high risk for experiencing homelessness a year following an ED visit.^21^ Evidence also shows that transportation barriers are high among those who present to the ED.^22^

As any increase in ED utilization represents a corresponding increase in associated costs, there is an urgent need to understand the underlying social drivers of ED utilization to appropriately develop targeted interventions to account for social risk burden and to decrease ED utilization and improve population health. While existing data shows that individual social risk factors are linked to increased ED utilization, little has been done to understand the prevalence of multiple social risk factors and their association with ED utilization at a national level. The purpose of this study, therefore, is to examine the relationship between multiple social risk factors (economic instability, lack of community, limited educational attainment, food insecurity, social isolation, and limited access to care) and ED utilization in a nationally representative sample of US adults.

## METHODS

### Data Source

We used the National Health Interview Survey (NHIS), person, adult, and family person files. The NHIS gathers information from people across the US. Information gathered includes disease and conditions information as well as healthcare information.^23^

### Measures

The primary independent variables include the social risk factor domains initially described by Wray et al in 2022.^24^ These domains were treated as binary: having a positive response to any item in a domain was considered a ‘1’ while having no positive responses as ‘0.’ (Missing was defined as missing all items in a domain.) The prompts and criteria for each domain are listed in Table 2. The outcome was binary and defined as having any visits to the ED in the prior year (0 visits vs ≥1 visits). We also included the following covariates: age; race/ethnicity; insurance status; obesity; mental health (depression/anxiety); and comorbidities (hypertension, coronary heart disease, heart attack, stroke, asthma, ulcer, cancer, emphysema/chronic obstructive pulmonary disease, kidney disease, diabetes, liver disease, arthritis, migraine, and chronic pain).

**Table 2.** Characteristics of social determinants of health domains in adults, National Health Interview Survey, 2016–2018.

	Total sample (N = 238,888,238) (n = 82,364)
**Economic instability**	73.5%
Welfare assistance	1.2%
Income from state/county welfare	0.8%
Unemployed	37.5%
Ever applied for Social Security Disability insurance	7.7%
Subsidized rent	3.2%
Worry about maintaining current standard of living	36.1%
Worry about enough money for retirement	44.2%
Worry about paying normal monthly bills	26.5%
Worry about inability to pay rent, mortgage, or housing costs	21.0%
Worry about making minimum payment on credit cards	11.7%
**Lack of community**	40.5%
People in your neighborhood do not help each other out	16.6%
There are no people you can count on in your neighborhood	17.5%
People in your neighborhood cannot be trusted	16.1%
Do not live in a close-knit neighborhood	35.6%
Educational deficit	37.3%
No college or graduate degree	36.3%
English not well spoken	5.4%
**Food insecurity**	23.5%
Lose weight because not enough money for food	2.0%
Cut size of meals or skip meals in the past month	5.4%
Eat less than you should because not enough money for food	5.6%
Ever hungry but did not eat because no money for food	3.4%
Ever receive food stamps/SNAP in past year	12.1%
Worried that food would run out	12.6%
Food did not last until you could buy more	10.8%
Did not eat balanced meals due to costs	9.9%
Received benefits or food subsidies from WIC program	4.3%
**Social isolation**	27.2%
Lives alone	18.0%
Difficult to participate in social activities	8.4%
Difficulty going to events	9.9%
Delayed getting medical care due to lack of transportation	2.0%
**Inadequate access to care**	13.1%
Lacks regular place to go to when sick or need health advice	13.1%

*SNAP*, Supplemental Nutrition Assistance Program; *WIC*, Women, Infants and Children Program.

### Analyses

We compared sample demographics, reported as percentages and means, using chi-square and analysis of variance statistics. Logistic regression models were used to assess the unadjusted independent association between ED use as a binary variable and each of the six social risk domains, followed by domain-adjusted models (ie, simultaneously controlling for all six social risks). Then, we used the fully adjusted logistic regression models to evaluate the independent association between ED use as a binary variable and each of the six social risk domains, adjusting for covariates, including age, race/ethnicity, insurance status, obesity, mental health, and comorbidities. Covariates were selected for inclusion in the model based on prior evidence on the relevance of these variables as well as based on *P* = <0.25 in bivariate analyses. We performed statistical analysis with R v 4.0.3 (R Foundation for Statistical Computing, Vienna, Austria). To account for the complex survey design and produce population level estimates, weighting was done using the svydesign function in R. Statistical significance was set at *P* = < 0.05.

## RESULTS

The study sample was comprised of 82,364 individuals in the 2016–2018 period, which represents 238,888,238 adults in the US population. This time frame was selected for its robust set of social risk factors available in the dataset. Table 1 shows demographic characteristics of our study population. Almost 20% reported using the ED one or more times. Middle-aged adults (ages 40-64) accounted for about 42% of this sample, with young adults (ages 18-39) representing 38%. Older adults (65+) account for approximately 20% of the study sample. The majority (64.8%) identified as non-Hispanic White, with Hispanic accounting for the second-highest group (16.1%), followed by non-Hispanic Blacks at 12%, and non-Hispanic other at 7.2%. About 9% of the adults were uninsured. Approximately 42% had 1-2 comorbidities and 23.2% had three or more, with chronic pain (34%) and hypertension (31%) representing the two most common comorbidities.

**Table 1.** Characteristics of adults, National Health Interview Survey, 2016–2018.

	Total sample (N = 238,888,238) (n = 82,364)
ED visits (binary)	
0	80.2%
1+	19.8%
Age	
18–39	38.0%
40–49	16.3%
50–64	25.6%
65–74	12.0%
75+	8.1%
Sex	
Male	48.3%
Female	51.7%
Race/ethnicity	
Non-Hispanic White	64.8%
Non-Hispanic Black	12.0%
Non-Hispanic other	7.2%
Hispanic	16.1%
Health insurance	90.6%
Obesity	30.8%
Mental health issue	2.7%
Comorbidities	
Hypertension	31.1%
Coronary heart disease	4.5%
Heart attack	3.1%
Stroke	3.1%
Asthma	13.6%
Ulcer	6.1%
Cancer	9.4%
Emphysema/COPD	3.7%
Kidney disease	2.1%
Diabetes	9.9%
Liver disease	1.9%
Arthritis	23.8%
Migraine	15.2%
Chronic pain	34.3%
Comorbidity count	
0	34.3%
1-2	41.8%
3-4	17.5%
5+	5.7%

*COPD*, chronic obstructive pulmonary disease;*ED*, emergency department.

Table 2 shows characteristics of social risk domains in our study population. A total of 73.5% reported economic instability, 40.5% reported lack of community, 23.5% reported food insecurity, 27.2% reported social isolation, and 13.1% reported inadequate access to care.

Table 3 displays unadjusted, domain-adjusted (controlling for only social risks), and fully adjusted models (controlling of social risks, comorbidities, and all other covariates outlined in the table). All social risk factors (economic instability, lack of community, educational deficit, food insecurity, social isolation, and inadequate access to care) were significantly associated with higher odds of ED visits in the unadjusted models, with food insecurity, economic instability, and social isolation among those with higher odds ratios (OR) (OR 2.46, 95% confidence interval [CI] 2.32-2.59; OR 2.10, 95% CI 1.99-2.22; and OR 2.02, 95% CI 1.94-2.11, respectively). In the fully adjusted models, economic instability (OR 1.33, 95% CI 1.25-1.42), lack of community (OR 1.10, 95% CI 1.05-1.15), educational deficit (OR 1.12, 95% CI 1.06-1.18), food insecurity (OR 1.77, 95% CI 1.66-1.89), and social isolation (OR 1.32, 95% CI 1.26-1.39) were associated with higher odds of ED utilization. However, inadequate access to care was negatively associated with ED use in both adjusted models (fully adjusted: OR 0.75, 95% CI 0.69-0.81).

**Table 3.** Logistic regression for binary emergency department visits.

	Total sample		
	Unadjusted	Domain adjusted	Fully adjusted
Economic instability	2.10 (1.99, 2.22)^***^	1.61 (1.52, 1.71)^***^	1.33 (1.25, 1.42)^***^
Lack of community	1.33 (1.28, 1.39)^***^	1.17 (1.12, 1.23)^***^	1.10 (1.05, 1.15)^***^
Educational deficit	1.42 (1.36, 1.49)^***^	1.12 (1.07, 1.17)^***^	1.12 (1.06, 1.18)^***^
Food insecurity	2.46 (2.32, 2.59)^***^	2.10 (1.98, 2.23)^***^	1.77 (1.66, 1.89)^***^
Social isolation	2.02 (1.94, 2.11)^***^	1.76 (1.68, 1.84)^***^	1.32 (1.26, 1.39)^***^
Inadequate access to care	0.68 (0.63, 0.73)^***^	0.62 (0.57, 0.67)^***^	0.75 (0.69, 0.81)^***^
Age			
18–39 (ref)	-	-	-
40–49	-	-	0.72 (0.66, 0.77)^***^
50–64	-	-	0.58 (0.54, 0.63)^***^
65–74	-	-	0.58 (0.53, 0.63)^***^
75+	-	-	0.78 (0.71, 0.86)^***^
Sex (male)	-	-	0.85 (0.81, 0.89)^***^
Race/ethnicity			
Hispanic (ref)	-	-	-
Non-Hispanic White	-	-	1.10 (1.01, 1.20)^*^
Non-Hispanic Black	-	-	1.36 (1.23, 1.50)^***^
Non-Hispanic other	-	-	0.87 (0.77, 0.99)^*^
Health insurance (uninsured)	-	-	0.98 (0.89, 1.09)
Obesity	-	-	1.09 (1.03, 1.15)^**^
Mental health issue	-	-	1.31 (1.15, 1.49)^***^
Hypertension	-	-	1.32 (1.24, 1.40)^***^
Coronary heart disease	-	-	1.33 (1.19, 1.48)^***^
Heart attack	-	-	1.44 (1.27, 1.64)^***^
Stroke	-	-	1.73 (1.54, 1.93)^***^
Asthma	-	-	1.28 (1.20, 1.37)^***^
Ulcer	-	-	1.41 (1.29, 1.54)^***^
Cancer	-	-	1.26 (1.16, 1.35)^***^
Emphysema/COPD	-	-	1.55 (1.40, 1.72)^***^
Kidney disease	-	-	1.81 (1.59, 2.07)^***^
Diabetes	-	-	1.24 (1.15, 1.34)^***^
Liver disease	-	-	1.78 (1.51, 2.10)^***^
Arthritis	-	-	1.23 (1.16, 1.31)^***^
Migraine	-	-	1.54 (1.45, 1.64)^***^
Chronic pain	-	-	1.23 (1.16, 1.30)^***^

* *P* = < 0.05, **P = < 0.01, ***P = < 0.001.

*COPD*, chronic obstructive pulmonary disease; *ED*, emergency department.

## DISCUSSION

Overall, ∼20% of US adults had at least one ED visit, and social risk factors were highly prevalent in the study sample with 74% having economic instability, 41% reporting lack of community, 37% reporting educational deficits, 27% reporting social isolation, 24% reporting food insecurity, and 13% reporting inadequate access to care. In addition, fully adjusted models showed that economic instability, lack of community, educational deficits, food insecurity, and social isolation were independently associated with increased odds of ED visits, while inadequate access to care was significantly associated with lower odds of ED visits. This is one of the first studies to our knowledge that has assessed the relationship between multiple domains of social risk factors and ED utilization in a nationally representative US adult population.

Our findings are consistent with existing literature on the association of social risks with ED utilization. For example, studies by Estrella^25^ and Dean^26^ show food insecurity is significantly associated with increased ED use and ED expenditure even after adjustment for potential confounders.^25^ ^,^ ^26^ Seim^16^ has shown that economic instability and community factors, through neighborhood poverty, are positively associated with 9-1-1 ambulance utilization, a surrogate for ED utilization.^16^ In another study, Ku^27^ provides evidence that frequent users of the ED may be disproportionately homeless.^27^ Similarly, available literature highlights the relationship between social isolation and ED use. In an observational study of older patients, Mosen^26^ found that those who experience social isolation were more likely to have at least one ED visit than those who rarely or never experienced social isolation.^28^

The current findings show lack of access to care is negatively associated with ED use. Available evidence on the association between access to health and ED use is mixed.^29^ Evidence suggests that greater access to care can translate into greater receipt of preventative care and being more cognizant of diseases and health, resulting in increased use across ED and primary care visits.^30^ Conversely, lack of access can result in lower use of the ED, as the current findings show. On the contrary, some evidence shows that an increase in access to outpatient care is associated with a decrease in ED use.^29^ Given the mixed findings, there remains an urgent need for further evidence on how the presence or lack of access impacts ED utilization across populations.

Overall, this study provides new evidence for understanding the relationship between social risk factors and ED use for adults at the national level with implications across research, practice, and policy. Specifically, available evidence shows ED visit rates are higher for patients in lower-income and socially vulnerable communities, highlighting the need for specific initiatives aimed at understanding the drivers of their increased ED use, and the need to pay close attention to social risks and effective ways to address them.^10^ Federal efforts to reduce ED overuse currently focus on improving primary care;^10^ however, initiatives that have looked to increase the availability of low-cost options for the patients seeking these services, typically of low acuity, have yielded little in terms of reducing costs.^31^ ^,^ ^32^ While our study underscores the linkage of individual social risks with ED utilization, it also highlights a greater opportunity to reduce costs by addressing social risks. Further research can elucidate whether addressing each of these social risks will translate into decreased ED use and cost.

Although various professional organizations recommend that health systems and clinicians incorporate social determinants of health and social risk screening into care models,^10^ ^,^ ^11^ a vast majority of healthcare systems and hospitals (ie, 70%) do not have dedicated funds to address social needs, with many health systems lacking community-level social needs data to inform investment.^33^ ^,^ ^34^ Even when a social risk is identified, emergency clinicians may not be aware of local resources or find it hard to best address it.^25^ Doran and colleagues developed a screening tool for ED patients to identify the risk of becoming homeless to refer for services and support for homelessness prevention^9^; tools such as these, using models for referral services,^35^ are greatly needed to assess across the spectrum of social risk factors known to impact health and lead to additional ED utilization.^9^ While our study adds to the evidence on the role of social risks on ED utilization, there is need for research investigating how each risk is driving ED use, what initiatives can be taken by communities and policymakers to reduce such risks, and how EDs can better accommodate the patients who are experiencing these risks, both to reduce costs and ED burden, and also to improve their health outcomes.

Physicians can look to social emergency medicine (EM), an emerging field within EM, as a path forward to account for the intersection between emergency care and social determinants of health.^36^ Social EM emphasizes a more holistic care model in the ED to better serve the populations who frequently visit the ED and receive care without adequate understanding by clinicians of the social forces at play. Our findings support the importance of this evolving field as a promising platform in mitigating the social risk burden on a broader scale and reducing ED utilization, especially among socially vulnerable populations.^37^

## LIMITATIONS

There are some limitations that must be considered while interpretating our study findings. Although our study is based on a nationally representative sample, it excluded institutionalized individuals; therefore, the finding may not generalize to that segment of the US population. Secondly, to maintain a robust set of social risk factors, the dataset included NHIS data prior to the 2019 revision. For this reason, this study does not capture additional social risk factors that may have developed as a result of the COVID-19 pandemic. In addition, while our models controlled for relevant confounding variables, we did not have data on all possible confounders, which may have biased our estimates. Also, since the responses to all survey questions are based on self-report, they are subject to recall bias. Finally, given that the study is cross-sectional, we cannot speak to causality.

## CONCLUSION

This study of a nationally representative sample of adults indicates that social risk factors are significantly associated with ED utilization. Specifically, economic instability, lack of community, educational deficit, food insecurity, and social isolation are associated with higher odds of ED use, whereas inadequate access to care is associated with lower ED use in fully adjusted models. Further research is needed to better understand potential pathways and mechanisms that underlie these associations. Interventions that can effectively address social risks have a potential to reduce unnecessary ED utilization and reduce healthcare costs. Emphasis should be placed on building infrastructure for screening and prevention programs for handoffs and referrals.
